# Supplementary material for: Maternal Dietary Patterns Are Associated with Pre-Pregnancy Body Mass Index and Gestational Weight Gain: Results from the “Mamma & Bambino” Cohort
Source: Nutrients. 2019 Jun 10;11(6):1308. doi: 10.3390/nu11061308 (PMC6627583; doi:10.3390/nu11061308)
Supplement: Supplementary file 1 [file nutrients-11-01308-s001.pdf]

Maternal Dietary Patterns Are Associated with Pre-Pregnancy Body Mass Index and Gestational Weight Gain: Results from the "Mamma &Bambino" Cohort

Andrea Maugeri[1], Martina Barchitta[1], Giuliana Favara[1], Maria Clara La Rosa[1], Claudia La Mastra[1], Roberta Magnano San Lio[1], Antonella Agodi[1*]

[1] Department of Medical and Surgical Sciences and Advanced Technologies "GF Ingrassia", University of Catania, Catania, Italy, Via S. Sofia 87, 95123 Catania, Italy
* Correspondence: agodia@unict.it (A.A.)

Supplementary Table 1. Food grouping used in the dietary pattern analysis.

95 FFQ Food Items	39 Food Groups
Spirits and other alcoholic drinks	Alcoholic drinks
Beer	Beer
Butter and margarine	Butter and Margarine
Canned fish	Canned fish
Cereals	Cereals
Coffee	Coffee
Cooked vegatables	Cooked vegetables
Spinach	
Carrots	
Peppers	
Aubergine	
Cauliflower, broccoli	
Zucchini, pumpkin	
Artichoke	
Mushroom	
Ketchup	Dipping sauces
Mayonnaise	
Eggs	Eggs
Fish (high in fat)	Fish
Fish (medium in fat)	
Fish (low in fat)	
Fries	Fries
Apple	Fruit
Pear	

Banana	
Peach, nectarine, prune	
Apricot	
Orange, mandarin	
Strawberries	
Melon, mango	
Watermelon	
White grape	
Red grape	
Pineapple	
Cherries	
Kiwi	
Multivitamin juice	Fruit juice
Pineapple juice	
Orange juice	
Pear juice	
Peach juice	
Fruit salad	Fruit salad
Hard cheeses	Hard cheese
Legumes	Legumes
Green bean	
Peas	
Whole milk	Milk
Semi-skimmed milk	
Skimmed milk	
Nuts	Nuts
Offal	Offal
Olive oil	Olive oil
Pasta	Pasta
Pizza	Pizza
Boiled potatoes	Potatoes
Salami	Processed meat
Ham (cooked or uncooked)	
Bresaola	
Fesa, chicken breast	
Wurstel pork	
Wurstel chicken	
Mortadella	
Green salad	Raw vegetable
Fresh tomatoes	
Fennel	
Beef, veal, lamb meat	Red meat

Pork meat	
Horse meat	
Rice	Rice
Popcorn, pretzels	Salty snacks
Wrapped potato chips	
Shellfish	Shellfish
Soft cheeses	Soft cheese
Soup	Soup
Cake, pastries	Sweets
Ice cream	
Pastry cream, pudding	
Chocolate, snack chocolate	
Candy	
Jam, marmalade	
Tea	Tea
Vegetable oil	Vegetable oil
Brioches	White bread
Cookies	
Bread	
Rusks, crackers	
White meat	White meat
Wholemeal biscuits	Wholemeal bread
Wholemeal bread	
Rusks, wholemeal crackers	
Red wine	Wine
Rosè wine, white wine	
Full fat yoghurt	Yoghurt
Low fat yoghurt	
Water	Not applicable

Supplementary Table 2. Factor loadings which characterized dietary patterns in two randomly selected subgroups (n = 100) from the "Mamma and Bambino" cohort.

Food groups	Factor Loadings			
	1st sub-sample (n = 100)		2nd sub-sample (n = 100)	
	PCA1	PCA2	PCA1	PCA2
**White bread**	**0.200**	0.112	0.170	0.182
**Whole-meal bread**	-0.196	0.184	-0.199	0.085
**Cereals**	-0.180	0.185	0.010	0.195
**Butter and Margarine**	0.136	-0.041	0.046	-0.101
**Milk**	0.139	0.189	0.189	-0.089
**Yogurt**	-0.184	0.193	-0.064	0.173
**Olive oil**	0.186	0.087	0.086	0.187
**Vegetable oil**	0.182	-0.093	0.134	-0.191
**Soft cheese**	0.065	0.075	0.095	0.125
**Hard cheese**	0.192	0.136	0.102	0.163
**Eggs**	0.120	0.174	0.020	0.186
**Processed meat**	0.195	-0.199	0.174	**-0.201**
**Red meat**	**0.509**	0.086	**0.534**	-0.036
**White meat**	-0.130	0.187	0.080	0.108
**Offal**	0.024	0.116	0.064	0.102
**Fish**	-0.191	0.195	-0.198	0.200
**Shellfish**	0.076	0.153	-0.066	0.187
**Canned fish**	0.017	0.140	0.024	0.100
**Fruit**	-0.150	0.197	-0.190	**0.201**
**Fruit salad**	-0.044	0.165	-0.084	0.135
**Raw vegetable**	-0.177	0.190	-0.193	0.188
**Cooked vegetable**	-0.192	**0.685**	**-0.200**	**0.775**
**Legumes**	-0.079	**0.572**	-0.188	**0.492**
**Soup**	-0.198	**0.490**	-0.178	**0.520**
**Potatoes**	-0.098	**0.261**	-0.140	**0.321**
**Fries**	**0.776**	-0.109	**0.807**	**-0.203**
**Rice**	-0.147	0.182	-0.187	0.154
**Pasta**	0.198	0.052	**0.203**	0.082

**Pizza**	-0.011	**0.423**	0.021	**0.233**
**Nuts**	0.022	0.163	0.104	0.104
**Sweets**	0.195	0.012	**0.205**	0.078
**Salty snacks**	**0.661**	0.132	**0.592**	0.178
**Dipping sauces**	**0.806**	0.183	**0.789**	0.056
**Wine**	0.047	0.194	0.079	0.096
**Beer**	0.108	-0.035	0.124	0.076
**Alcoholic drinks**	**0.709**	0.167	**0.724**	0.109
**Coffee**	0.149	0.153	0.178	0.124
**Tea**	-0.148	-0.130	-0.190	-0.093
**Fruit juice**	0.095	0.093	0.109	0.089

Factor loadings characterizing each dietary pattern (absolute value ≥0.2) are indicated in bold font.

Supplementary Figure 1. Scree plot of the eigenvalues.

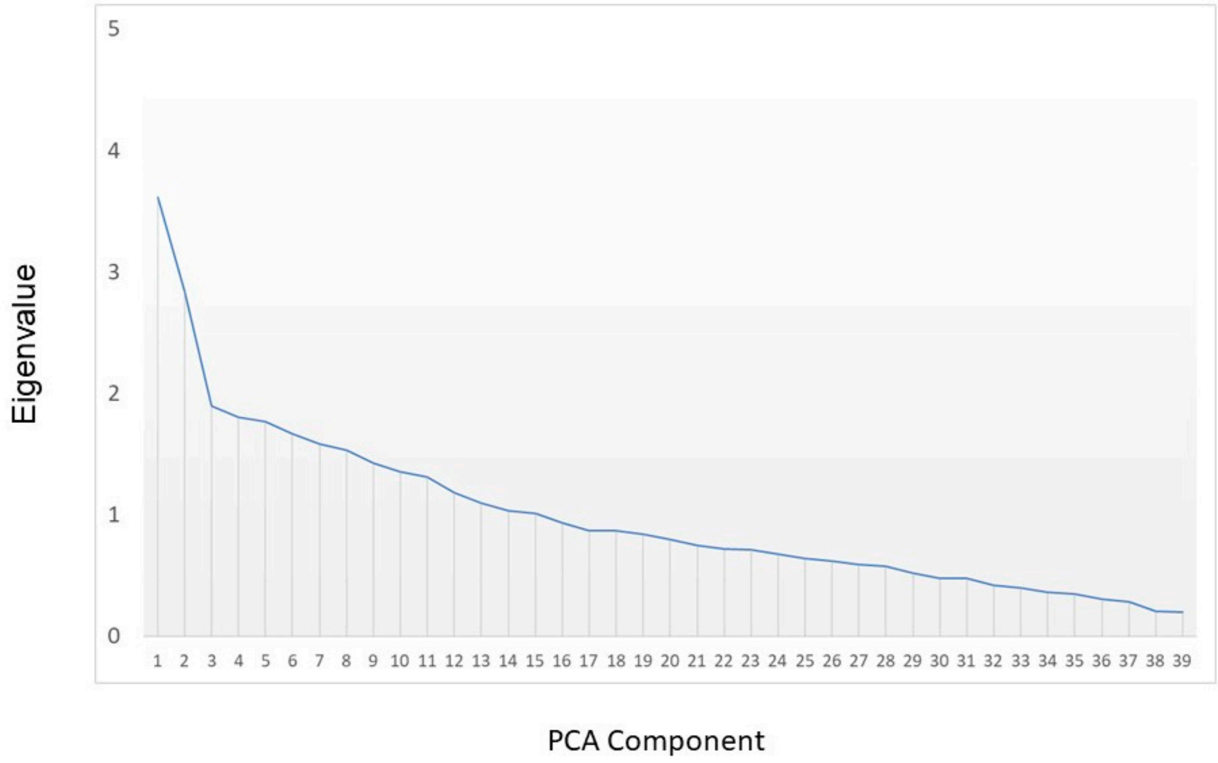

We used the scree plot examination to determine the appropriate number of dietary patterns. The scree plot represents the partitioning of the total variation (i.e., eigenvalue) accounted for each principal component, against the principal component number.

PCA: principal component analysis.
